# Two-sample fecal immunochemical testing as a tool to avert colonoscopy in symptomatic patients: a prospective multicenter cohort study

**DOI:** 10.1055/a-2650-0664

**Published:** 2025-08-25

**Authors:** Sarah Moen, Pieter H.A. Wisse, Fleur Marijnissen, Hannah Raab, Iris Lansdorp-Vogelaar, Jeroen M. Jansen, Merel M. Tielemans, I. Leeuwenburgh, Leonieke M.M. Wolters, Lieke Hol, Pieter C.J. Ter Borg, Frank C. Bekkering, Sanna Mulder, Ingrid Schot, Marieke Frasa, Marc Thelen, Anneke J. van Vuuren, Manon C.W. Spaander

**Affiliations:** 1 6993 Department of Gastroenterology and Hepatology, Erasmus MC University Medical Center Rotterdam, Rotterdam, Netherlands; 2 6993 Department of Public Health, Erasmus MC University Medical Center Rotterdam, Rotterdam, Netherlands; 3 Department of Gastroenterology and Hepatology, PoliDirect Independent Treatment Centers, Amsterdam, Netherlands; 4 37226 Department of Gastroenterology and Hepatology, Bravis Hospital, Roosendaal, Netherlands; 5 Department of Gastroenterology and Hepatology, Franciscus Gasthuis and Vlietland, Rotterdam, Netherlands; 6 Department of Gastroenterology and Hepatology, Albert Schweitzer Hospital, Dordrecht, Netherlands; 7 7000 Department of Gastroenterology and Hepatology, Maasstad Hospital, Rotterdam, Netherlands; 8 36863 Department of Gastroenterology and Hepatology, Ikazia Hospital, Rotterdam, Netherlands; 9 Department of Gastroenterology and Hepatology, DC Clinics Independent Treatment Centers, Capelle aan den IJssel, Netherlands; 10 84744 Department of Gastroenterology and Hepatology, Reinier de Graaf Gasthuis, Delft, Netherlands; 11 10192 Department of Gastroenterology and hepatology, IJsselland Hospital, Capelle aan den IJssel, Netherlands; 12 10233 Department of Clinical Chemistry, Jeroen Bosch Hospital, 's-Hertogenbosch, Netherlands; 13 6034 Department of Laboratory Medicine, Radboud University Medical Center, Nijmegen, Netherlands; 14 SKML, EQA Organization for Laboratory Medicine, Nijmegen, Netherlands

## Abstract

**Background:**

In most colonoscopies performed for bowel symptoms, no significant lesions are found. To decrease the number of unnecessary colonoscopies, we evaluated the performance of two-sample fecal immunochemical testing (FIT) in ruling out significant lesions.

**Methods:**

Symptomatic patients referred for colonoscopy were instructed to perform two FITs from separate bowel movements prior to colonoscopy. Colonoscopy and pathology data were collected. Two-sample FIT was considered positive when FIT1 and/or FIT2 results were positive. Sensitivity and negative predictive value (NPV) for advanced neoplasia, advanced serrated polyps, and colitis were determined at different cutoff values.

**Results:**

949 patients (median age 61 years, 50.6% male) from 10 centers were included. The highest NPVs and sensitivities were reached with two-sample FIT using the lowest limit of fecal hemoglobin detection (>1.7 µg Hb/g). For advanced neoplasia and CRC, this resulted in NPVs of 95.6% and 99.7%, and sensitivities of 71.7% and 93.9%, respectively. Sensitivity for advanced neoplasia was higher (84.6%) in patients with the alarm symptoms of rectal blood loss and/or anemia. NPV and sensitivity for inflammatory bowel disease were 99.3% and 83.3%, respectively. Concordant negative results were found for 675 patients (71.1%).

**Conclusions:**

Despite a high NPV, two-sample FIT still missed 28.3% of advanced neoplasia. Therefore, two-sample FIT may play a role in determining the need for colonoscopy in symptomatic patients, but it misses too many lesions to be used as the sole determinant for averting colonoscopy.

## Abstract (de)

**Graphical abstract:**

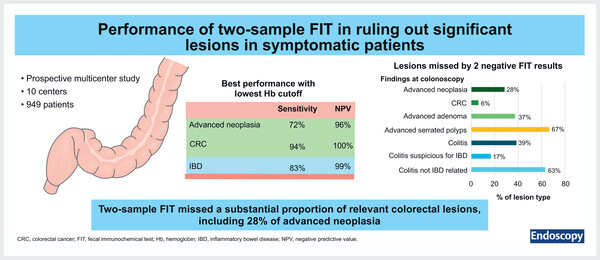

## Introduction

In patients with symptoms suggestive of colorectal lesions, colonoscopy is frequently performed. However, in most of these colonoscopies no relevant abnormalities are found. A noninvasive preselection tool to avert unnecessary colonoscopy would be beneficial to both the patient and the health care system by minimizing overuse of colonoscopy resources.

Fecal immunochemical testing (FIT) for human hemoglobin (Hb) is recommended as an effective tool in colorectal cancer (CRC) screening to identify those in need of subsequent colonoscopy 1 . Evidence suggests a role for FIT as a preselection tool in symptomatic patients as well. A meta-analysis conducted in symptomatic patients in primary health care showed that performance of one-sample FIT using any detectable hemoglobin as the cutoff level (2–7 µg/g feces) resulted in a high sensitivity of 93.4% for detecting CRC 2 . Based on these results, both the National Institute for Health and Care Excellence guidelines, as well as a joint guideline from the Association of Coloproctology of Great Britain and Ireland and the British Society of Gastroenterology, highlighted that FIT can be used as a rule-in test to triage patients with bowel symptoms who require fast-track referral 3 4 . However, when using FIT as a rule-out test for colonoscopy, other relevant lesions such as advanced adenoma are important as well. Another meta-analysis in symptomatic patients showed that FIT using 10 µg Hb/g feces as the cutoff level had a sensitivity of 92.1% for CRC alone; however, when including advanced adenoma, sensitivity dropped to 62.6% 5 .

When FIT is used as a rule-out test to avert colonoscopy, a high negative predictive value (NPV) and a high sensitivity for advanced neoplasia (both CRC and advanced adenoma) are important to limit the number of false negatives and thus missed lesions. This requirement justifies using the lowest possible cutoff level for FIT to increase sensitivity and NPV. This does lead to a lower specificity 2 , but that is less important in symptomatic patients who currently all undergo colonoscopy. Another solution to increase sensitivity and NPV for advanced neoplasia in symptomatic patients might be to perform two-sample FIT. Several studies have investigated the use of two FIT samples in symptomatic patients. Some studies included only two-sample FIT for CRC alone 6 7 . One study, however, comprising 2637 symptomatic patients who completed two-sample FIT, showed a sensitivity of 84.1% for CRC with one-sample FIT, which increased to 96.6% with two-sample FIT 8 . The increase in sensitivity was also found for advanced neoplasia: 64.4% with one-sample FIT, increasing to 81.6% with two-sample FIT 8 . This promising result was shown at a cutoff level of 10 µg Hb/g feces. Sensitivity might be even higher when a lower cutoff level is used; however, the study did not provide data with lower cutoff values. One study did include any detectable hemoglobin as the cutoff value, resulting in an increase in sensitivity for advanced neoplasia from 96.6% with one-sample FIT to 100% with two-sample FIT 9 . However, the generalizability of these results is limited, as the study was conducted in only 208 patients, 29 of whom were diagnosed with advanced neoplasia.

To reduce the number of unnecessary colonoscopies, we evaluated the performance of two-sample FIT in a multicenter cohort of symptomatic patients, using various hemoglobin cutoff values, including any detectable level.

## Methods

In this prospective multicenter cohort study, patients referred for colonoscopy because of anemia or symptoms were asked to perform two FITs from separate subsequent bowel movements prior to bowel preparation and colonoscopy.

Patients were approached between October 2019 and December 2021 in 10 participating centers, consisting of 8 hospitals and 2 endoscopy centers. Patients were eligible for inclusion when they were referred for diagnostic colonoscopy for symptoms or anemia. Patients were excluded if they were referred for diagnostic colonoscopy for other indications including screening or surveillance for polyps, CRC, inflammatory bowel disease (IBD), or hereditary CRC. Eligible patients were selected by their treating physician in the participating centers and were asked for permission to be approached by a member of the study team from the main study center. When consented, patients were contacted by phone to explain the study. When a patient was willing to participate, a package with two FITs and instructions was sent to their home address and an online questionnaire was sent out by email. The FITs were performed in two subsequent stool samples prior to bowel preparation and sent by patients to the main laboratory at the main study center. Patients underwent their colonoscopy at the participating center to which they were referred. Data from subsequent colonoscopy and pathology were collected. The FIT used was FOB Gold measured on a Sentifit 270 analyzer (Sentinel Diagnostics, Milan, Italy). Laboratory results were provided for both the FIT method and the FIT Wide method. For the FIT method, FOB Gold set H calibrators and controls were used, with a calibration measuring range of 10–840 ng/mL (equivalent to 1.7–142.8 ug/g). For the FIT Wide method, FOB Gold set W calibrators and controls were used, with a calibration measuring range of 15–1000 ng/mL (equivalent to 2.55–170 ug/g). Primary analyses were based on the FIT method H results. FIT method W results are provided in the Supplementary material to inform countries using the latter method. The study design was approved by the Institutional Review Board of Erasmus MC (registration number MEC-2019–0381) and by the review boards of the participating centers. All included patients provided written informed consent.

### Dataset

Advanced neoplasia was defined as histopathologically confirmed CRC and/or advanced adenoma. Advanced adenoma was defined as an adenoma ≥10 mm, and/or with high grade dysplasia, and/or with >25% villous histology. Advanced serrated polyp was defined as a traditional serrated adenoma or a sessile serrated lesion (SSL) with dysplasia, or a SSL ≥10 mm, or a hyperplastic polyp ≥10 mm. Colitis was categorized as “colitis suspicious for IBD” and “colitis not IBD related.” Two-sample FIT was considered positive when FIT1 and/or FIT2 was positive. One-sample FIT was considered positive when the first FIT was positive. Alarm symptoms were defined as rectal blood loss and/or anemia. These symptoms were selected as alarm symptoms based on a meta-analysis of studies reporting the diagnostic accuracy of alarm features for CRC, in which rectal blood loss and anemia were found to be the symptoms with the highest risk 10 . Presence of alarm symptoms was determined based on the clinician-defined indication for colonoscopy rather than the symptoms reported in patient questionnaires.

### Outcomes

We assessed the performance of two-sample FIT to rule out relevant lesions such as advanced neoplasia, advanced serrated polyp, and colitis in patients referred for diagnostic colonoscopy because of symptoms or anemia. Performance was determined in terms of sensitivity, specificity, positive predictive value (PPV), and NPV at different cutoff values for both one-sample and two-sample FIT.

The cutoff value with the highest NPV and sensitivity was selected for the main analyses. Focus on NPV and sensitivity, and thus a low proportion of false negatives, was chosen because the goal of the current study was to find a tool to rule out relevant lesions to avert colonoscopy in symptomatic patients. Given that all symptomatic patients currently undergo diagnostic colonoscopy, lower PPV and specificity, and thus a higher proportion of false positives, was accepted when a large proportion of colonoscopies could be averted.

The main analyses at the selected cutoff value included the performance of one-sample and two-sample FIT, including positive and negative likelihood ratios and evaluation of the proportion of patients with concordant negative results from two-sample FIT in whom colonoscopy could potentially be averted. We also assessed the presence of relevant lesions in each two-sample FIT outcome group consisting of either concordant negative, discordant, or concordant positive results. Performance of two-sample FIT in terms of sensitivity, specificity, PPV, and NPV, including positive and negative likelihood ratios, was also determined separately for patients with and without the alarm symptoms. For both groups with and without alarm symptoms, we assessed the presence of relevant lesions in each two-sample FIT outcome group consisting of either concordant negative, discordant, or concordant positive results.

Baseline characteristics of the patients were also gathered, including age and sex obtained from the electronic patient systems in the participating centers, as well as patient-reported medical history and symptoms derived from the questionnaires.

### Statistical analyses

Descriptive statistics were performed to assess the baseline characteristics, diagnostic yield of colonoscopy, and performance of two-sample and one-sample FIT in detecting or ruling out relevant lesions. Variables are presented as mean with SD or median with interquartile range (IQR) for numerical data, or as count with percentage for categorical data. The performance of two-sample and one-sample FIT in terms of sensitivity, specificity, PPV, and NPV are presented as percentages. Wilson score intervals were used to determine the 95%CIs of these percentages. Positive likelihood ratio was defined as sensitivity divided by 1–specificity, and negative likelihood ratio was defined as 1–sensitivity divided by specificity. A negative likelihood ratio <0.1 was considered to provide strong evidence for ruling out a diagnosis.

All analyses were performed using IBM SPSS version 25 (IBM Corp., Armonk, New York, USA).

## Results

**Fig. 1:**
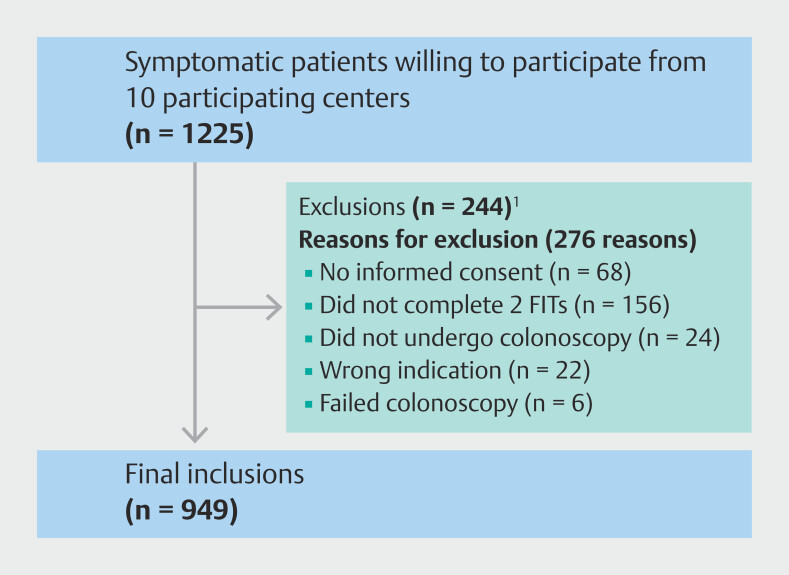
Inclusion flow chart. FIT, fecal immunochemical test. ^1^ Some patients had more than one reason for exclusion.

A total of 1225 patients referred for colonoscopy because of anemia or symptoms were willing to participate. A proportion of these patients were excluded for various reasons, including failure to provide written informed consent, failure to complete both FITs, cancellation or failure of the colonoscopy, or incorrect indication for colonoscopy. As a result, 949 patients performed two-sample FIT prior to their colonoscopy and were included in our trial. Of these patients, 930 also completed the corresponding questionnaire. The inclusion flow chart is shown in Fig. 1 .

Patients had a median age of 61 years (IQR 19 years) and 50.6% of them were male ( Table 1 ). The four most common patient-reported symptoms were abdominal pain (71.0%), changed stool pattern (64.1%), residual stool after toilet visit (59.9%), and rectal blood loss (42.2%). Indication for colonoscopy as defined by the clinician was anemia in 78 patients (8.2%), rectal blood loss in 358 patients (37.7%), changed stool pattern in 402 patients (42.4%), and other symptoms in 403 patients (42.5%) ( Table 2 ). Some patients had more than one indication for colonoscopy, for example abdominal pain and rectal blood loss.

Cecal intubation was reached in 95.9% of the colonoscopies and median Boston Bowel Preparation Score was 9. Diagnostic yield of colonoscopy was advanced neoplasia in 106 patients (11.2%), which included CRC in 33 patients (3.5%) and/or advanced adenoma in 75 patients (7.9%); 2 patients had both CRC and advanced adenoma. Advanced serrated polyps were found in 33 patients (3.5%) and included serrated adenoma in 4 patients (0.4%), and/or sessile serrated lesions (SSLs) with dysplasia in 1 patient (0.1%), and/or SSL ≥10 mm without dysplasia in 20 patients (2.1%), and/or hyperplastic polyp ≥10 mm in 8 patients (0.8%). Colitis was found in 57 patients (6.0%) and included colitis suspicious for IBD in 30 patients (3.2%) and colitis not IBD related in 27 patients (2.8%). Other lesions included nonadvanced adenoma in 255 patients (26.9%), SSL <10 mm without dysplasia in 41 patients (4.3%), and hyperplastic polyps <10 mm in 87 patients (9.2%).

**Table:** **Table 1** Baseline characteristics of symptomatic patients included in the trial.

Patient characteristics	
All patients (N = 949)	
Median age (IQR), years	61 (19)
Sex, male, n (%)	480 (50.6)
Patient-reported characteristics (N = 930)	
Bowel-related medical history, n (%)	
Polyps	49 (5.3)
CRC	6 (0.6)
IBD	8 (0.9)
IBS	59 (6.3)
Diverticulitis	35 (3.8)
Hemorrhoids	463 (49.8)
Anal fissure	238 (25.6)
Other bowel-related conditions ^1^	32 (3.4)
Other characteristics, n (%)	
Surgical removal of part of the bowel	30 (3.2)
CRC in family	255 (27.4)
(History of) smoking	450 (48.4)
Use of anticoagulant medication	158 (17)
Symptoms	
Rectal blood loss	392 (42.2)
Melena	141 (15.2)
Changed stool pattern	596 (64.1)
Abdominal pain	660 (71.0)
Anal pain	225 (24.2)
Incontinence	278 (29.9)
Residual stool after toilet visit	557 (59.9)
Mucus in stool	248 (26.7)
Fatigue	377 (40.5)
Unintentional weight loss	109 (11.7)
Abdominal mass	156 (16.8)
Anal mass	128 (13.8)
CRC, colorectal cancer; IBD, inflammatory bowel disease; IBS, irritable bowel syndrome; IQR, interquartile range. ^1^ Other bowel-related conditions included ileus, appendicitis, colitis, abscess, bacterial infection in stool, fistula, diverticula, prolapse, and lactose/gluten deficiency.	

**Table:** **Table 2** Colonoscopy characteristics and outcomes in symptomatic patients.

	N = 949
Indication for colonoscopy as defined by clinician ^1^ , n (%)	
Anemia	78 (8.2)
Rectal blood loss	358 (37.7)
Changed stool pattern	402 (42.4)
Other symptoms	403 (42.5)
Quality colonoscopy ^2^ , n (%)	
Cecal intubation, complete	910 (95.9)
BBPS, median (IQR)	9 (8–9)
Diagnostic yield colonoscopy ^3^ , n (%)	
Advanced neoplasia	106 (11.2)
CRC	33 (3.5)
Advanced adenoma ^4^	75 (7.9)
Advanced serrated polyps ^5^	33 (3.5)
Serrated adenoma	4 (0.4)
SSL with dysplasia	1 (0.1)
SSL ≥10 mm without dysplasia	20 (2.1)
Hyperplastic polyp ≥10 mm	8 (0.8)
Colitis	57 (6.0)
Colitis (suspicious for IBD)	30 (3.2)
Colitis (not IBD related)	27 (2.8)
Other lesions	
Nonadvanced adenoma	255 (26.9)
SSL <10 mm without dysplasia	41 (4.3)
Hyperplastic polyp <10 mm	87 (9.2)
BBPS, Boston Bowel Preparation Scale; CRC, colorectal cancer; IBD, inflammatory bowel disease; IBS, irritable bowel syndrome; IQR, interquartile range; SSL, sessile serrated lesion. ^1^ Some patients had more than one indication for colonoscopy. ^2^ Cecal intubation was known for 948 patients and BBPS score was known for 934 patients. ^3^ Diagnostic yield of colonoscopy was pathology based. ^4^ Advanced adenoma was defined as adenoma ≥10 mm, and/or high grade dysplasia, and/or >25% villous histology. ^5^ Advanced serrated polyp was defined as traditional serrated adenoma or sessile serrated lesion (SSL) with dysplasia, or SSL ≥10 mm, or hyperplastic polyp ≥10 mm. For 17 SSLs without dysplasia and 44 hyperplastic polyps, size was unknown and lesions were classified as <10 mm, which could in theory have been advanced serrated polyps.	

### Performance of two-sample FIT in detecting relevant lesions

The performance of two-sample FIT and one-sample FIT in ruling out relevant lesions was determined at different cutoff values ( **Table 1s** ). Increasing the cutoff value led to an increase in PPV and specificity for all lesions, but a decrease in NPV and sensitivity. However, for CRC, NPV and sensitivity remained the same, whereas PPV and specificity increased when a cutoff value of 10 µg Hb/g was used instead of any detectable hemoglobin.

Results using FIT method W instead of FIT method H at different cutoff values are shown in **Table 2s** .

The highest NPV and sensitivity for all relevant lesions were found with two-sample FIT using any detectable hemoglobin level (>1.7 µg/g) as the cutoff value, and therefore this cutoff value was chosen for the main analyses ( Table 3 ). For advanced neoplasia, NPV was 94.7% (95%CI 92.8–96.1) with one FIT, increasing to 95.6% (95%CI 93.8–96.9) with the second FIT. Sensitivity for advanced neoplasia was 63.2% (95%CI 53.7–71.8) with one FIT, increasing to 71.7% (95%CI 62.5–79.4) with the second FIT. When looking at the detection of CRC specifically, two-sample FIT resulted in a sensitivity of 93.9% (95%CI 80.3–98.3) and an NPV of 99.7% (95%CI 98.9–99.9). For advanced serrated polyps, NPV was 96.6% (95%CI 95.0–97.7) with one FIT, which increased slightly to 96.7% (95%CI 95.1–97.8) with the second FIT. Sensitivity for advanced serrated polyps was 24.2% (95%CI 12.8–41.0) with one FIT, increasing to 33.3% (95%CI 19.7–50.4) with the second FIT. For colitis, NPV was 96.3% (95%CI 94.7–97.4) with one FIT, which increased slightly to 96.7% (95%CI 95.1–97.8) with the second FIT. Sensitivity for colitis was 52.6% (95%CI 39.9–65.0) with one FIT, increasing to 61.4% (95%CI 48.4–72.9) with the second FIT. Sensitivity was higher when looking at those with colitis suspicious for IBD: 76.7% (95%CI 59.1–88.2) for one FIT, increasing to 83.3% (95%CI 66.4–92.6) with the second FIT. For CRC, the negative likelihood ratio of one-sample FIT was 0.15, which improved to 0.08 (<0.1) with two-sample FIT. For all other lesions, negative likelihood ratios were higher than 0.1 for both one-sample and two-sample FIT.

**Table:** **Table 3** Performance of two-sample versus one-sample fecal immunochemical test in detecting relevant lesions using any detectable hemoglobin ^1^ as the cutoff value.

	Sensitivity, % (95%CI)	Specificity, % (95%CI)	PPV, % (95%CI)	NPV, % (95%CI)	+LR	–LR
Two-sample FIT ^2^						
Advanced neoplasia	71.7 (62.5–79.4)	76.5 (73.5–79.2)	27.7 (22.7–33.3)	95.6 (93.8–96.9)	3.05	0.37
CRC	93.9 (80.3–98.3)	73.5 (70.5–76.3)	11.3 (8.1–15.6)	99.7 (98.9–99.9)	3.54	0.08
Advanced adenoma	62.7 (51.4–72.8)	74.0 (71.0–76.8)	17.2 (13.2–22.1)	95.9 (94.1–97.2)	2.41	0.50
Advanced serrated polyps	33.3 (19.7–50.4)	71.3 (68.3–74.1)	4.0 (2.3–7.0)	96.7 (95.1–97.8)	1.16	0.94
Colitis	61.4 (48.4–72.9)	73.2 (70.2–76.0)	12.8 (9.4–17.3)	96.7 (95.1–97.8)	2.29	0.53
Colitis suspicious for IBD	83.3 (66.4–92.6)	72.9 (69.9–75.7)	9.1 (6.2–13.1)	99.3 (98.3–99.7)	3.07	0.23
Colitis not IBD related	37.0 (21.5–55.7)	71.4 (68.4–74.2)	3.6 (2.0–6.5)	97.5 (96.0–98.4)	1.29	0.88
One-sample FIT						
Advanced neoplasia	63.2 (53.7–71.8)	82.6 (79.9–85.0)	31.3 (25.5–37.8)	94.7 (92.8–96.1)	3.63	0.45
CRC	87.9 (72.7–95.2)	79.8 (77.1–82.3)	13.6 (9.6–18.8)	99.5 (98.7–99.8)	4.35	0.15
Advanced adenoma	53.3 (42.1–64.2)	80.1 (77.3–82.6)	18.7 (14.0–24.5)	95.2 (93.4–96.5)	2.68	0.58
Advanced serrated polyps	24.2 (12.8–41.0)	77.5 (74.7–80.1)	3.7 (2.0–7.2)	96.6 (95.0–97.7)	1.08	0.98
Colitis	52.6 (39.9–65.0)	79.4 (76.6–81.9)	14.0% (10.0–19.3)	96.3 (94.7–97.4)	2.55	0.60
Colitis suspicious for IBD	76.7 (59.1–88.2)	79.2 (76.5–81.7)	10.7 (7.2–15.6)	99.0 (98.0–99.5)	3.69	0.29
Colitis not IBD related	25.9 (13.2–44.7)	77.5 (74.7–80.1)	3.3 (1.6–6.6)	97.3 (95.9–98.2)	1.15	0.96
CRC, colorectal cancer; FIT, fecal immunochemical test; IBD, inflammatory bowel disease; LR, likelihood ratio; NPV, negative predictive value; PPV, positive predictive value. ^1^ Any detectable hemoglobin corresponds to the lower limit of detection of 1.7 µg Hb/g feces. ^2^ Two-sample FIT was classified as positive when FIT1 and/or FIT2 was positive.						

Sensitivity for advanced neoplasia using two-sample FIT with any detectable hemoglobin as the cutoff value was higher in the subgroup of patients with alarm symptoms (84.6%, 95%CI 73.9–91.4) compared with both the whole study population (71.7%, 95%CI 62.5–79.4) and the subgroup of patients without alarm symptoms (51.2%, 95%CI 36.5–65.8) ( Table 4 ). NPV was relatively high in all groups. A negative likelihood ratio <0.1 was only observed for CRC in the subgroup with alarm symptoms.

**Table:** **Table 4** Performance of two-sample fecal immunochemical test in detecting relevant lesions using any detectable hemoglobin ^1^ as the cutoff value in patients with and without alarm symptoms.

	N	Two-sample FIT ^2^					
		Sensitivity, % (95%CI)	Specificity, % (95%CI)	PPV, % (95%CI)	NPV, % (95%CI)	+LR	–LR
With alarm symptoms	423						
Advanced neoplasia	65	84.6 (73.9–91.4)	69.8 (64.9–74.4)	33.7 (26.9–41.3)	96.2 (93.1–97.9)	2.80	0.22
CRC	26	96.2 (81.1–99.3)	65.2 (60.4–69.8)	15.3 (10.6–21.7)	99.6 (97.9–99.9)	2.76	0.06
Advanced adenoma	41	78.0 (63.3–88.0)	65.7 (60.8–70.3)	19.6 (14.3–26.4)	96.5 (93.6–98.2)	2.27	0.33
Advanced serrated polyps	17	35.3 (17.3–58.7)	61.3 (56.5–65.9)	3.7 (1.7–7.8)	95.8 (92.6–97.6)	0.91	1.06
Colitis	31	67.7 (50.1–81.4)	63.8 (58.9–68.4)	12.9 (8.6–18.9)	96.2 (93.1–97.9)	1.87	0.51
Colitis suspicious for IBD	22	86.4 (66.7–95.3)	64.1 (59.3–68.6)	11.7 (7.6–17.5)	98.8 (96.7–99.6)	2.41	0.21
Colitis not IBD related	9	22.2 (6.3–54.7)	61.1 (56.3–65.7)	1.2 (0.3–4.4)	97.3 (94.6–98.7)	0.57	1.27
Without alarm symptoms	526						
Advanced neoplasia	41	51.2 (36.5–65.8)	81.4 (77.7–84.7)	18.9 (12.7–27.2)	95.2 (92.7–96.9)	2.75	0.60
CRC	7	85.7 (48.7–97.4)	79.8 (76.1–83.0)	5.4 (2.5–11.3)	99.8 (98.7–100)	4.24	0.18
Advanced adenoma	34	44.1 (28.9–60.6)	80.5 (76.8–83.8)	13.5 (8.4–21.1)	95.4 (93.0–97.1)	2.26	0.69
Advanced serrated polyps	16	31.3 (14.2–55.6)	79.2 (75.5–82.5)	4.5 (1.9–10.1)	97.3 (95.3–98.5)	1.51	0.87
Colitis	26	53.8 (35.5–71.2)	80.6 (76.9–83.8)	12.6 (7.6–20.1)	97.1 (95.0–98.3)	2.77	0.57
Colitis suspicious for IBD	8	75.0 (40.9–92.9)	79.7 (76.1–83.0)	5.4 (2.5–11.3)	99.5 (98.3–99.9)	3.70	0.31
Colitis not IBD related	18	44.4 (24.6–66.3)	79.7 (76.0–83.0)	7.2 (3.7–13.6)	97.6 (95.6–98.7)	2.19	0.70
CRC, colorectal cancer; FIT, fecal immunochemical test; IBD, inflammatory bowel disease; LR, likelihood ratio; NPV, negative predictive value; PPV, positive predictive value. ^1^ Any detectable hemoglobin corresponds to the lower limit of detection of 1.7 µg Hb/g feces. ^2^ Two-sample FIT was classified as positive when FIT1 and/or FIT2 was positive.							

Sensitivity and NPV for advanced neoplasia for both groups with and without alarm symptoms was increased by performing two-sample FIT compared with one-sample FIT ( **Table 3s** ). For CRC within the subgroup without alarm symptoms, no improvement in sensitivity and NPV was seen when applying two-sample FIT compared with one-sample FIT.

### Potential decrease in unnecessary colonoscopies

Colonoscopy could potentially be averted in 675 patients (71.1%) who had concordant negative results when applying two-sample FIT using the lowest limit of fecal hemoglobin detection. Discordant results were found in 124 patients (13.1%) and concordant positive results were found in 150 patients (15.8%).

A substantial proportion of relevant lesions was still detected in the concordant negative group ( Table 5 ). Two negative FITs missed 30 advanced neoplasia (28.3% of all advanced neoplasia), which were mostly advanced adenomas (28; 37.3% of all advanced adenomas) but also two CRCs (6.1% of all CRCs). Of the two missed CRCs, one was located in the ascending colon and the other was located in the descending colon. Two negative FITs missed a substantial proportion of advanced serrated polyps (22; 66.7% of all advanced serrated polyps). A total of 22 colitis lesions were missed by two negative FITs (38.6% of all colitis), which were mostly colitis not IBD related (17; 63.0% of all colitis not IBD related), but also five colitis suspicious for IBD (16.7% of all colitis suspicious for IBD). The largest proportion of missed lesions by two negative FITs was seen in the subgroup without alarm symptoms ( Table 6 ).

**Table:** **Table 5** Relevant lesions found during colonoscopy after two-sample fecal immunochemical tests ^1^ with concordant negative, discordant, and concordant positive findings.

Colonoscopy findings	All lesions in category	Results from 2× FIT, n (%) ^2^		
		Concordant negative (n = 675)	Discordant (n = 124)	Concordant positive (n = 150)
Advanced neoplasia	106	30 (28.3)	14 (13.2)	62 (58.5)
CRC	33	2 (6.1)	2 (6.1)	29 (87.9)
Advanced adenoma	75	28 (37.3)	12 (16.0)	35 (46.7)
Advanced serrated polyps	33	22 (66.7)	6 (18.2)	5 (15.2)
Colitis	57	22 (38.6)	7 (12.3)	28 (49.1)
Colitis suspicious for IBD	30	5 (16.7)	3 (10.0)	22 (73.3)
Colitis not IBD related	27	17 (63.0)	4 (14.8)	6 (22.2)
CRC, colorectal cancer; FIT, fecal immunochemical test; IBD, inflammatory bowel disease. ^1^ The cutoff for positivity was any detectable hemoglobin (>1.7 µg Hb/g feces). ^2^ Calculated as percentage of lesion type.				

**Table:** **Table 6** Relevant lesions found during colonoscopy after two-sample fecal immunochemical tests ^1^ with concordant negative, discordant, and concordant positive findings in patients with and without alarm symptoms.

Colonoscopy findings	Results from 2× FIT		
	Concordant negative	Discordant	Concordant positive
With alarm symptoms (n = 423)	260	65	98
Advanced neoplasia	10	8	47
CRC	1	2	23
Advanced adenoma	9	6	26
Advanced serrated polyps	11	1	5
Colitis	10	1	20
Colitis suspicious for IBD	3	1	18
Colitis not IBD related	7	0	2
Without alarm symptoms (n = 526)	415	59	52
Advanced neoplasia	20	6	15
CRC	1	0	6
Advanced adenoma	19	6	9
Advanced serrated polyps	11	5	0
Colitis	12	6	8
Colitis suspicious for IBD	2	2	4
Colitis not IBD related	10	4	4
CRC, colorectal cancer; FIT, fecal immunochemical test; IBD, inflammatory bowel disease. ^1^ The cutoff for positivity was any detectable hemoglobin (>1.7 µg Hb/g feces).			

### Numbers needed to scope

When looking at the number needed to scope in the whole study population, 29 colonoscopies were needed to find one CRC ( **Table 4s** ). This number was higher among patients without alarm symptoms, where 75 colonoscopies were needed to detect one case of CRC. In addition, the number needed to scope to find one CRC was substantially higher in the subgroup with concordant negative FIT results, at 338 colonoscopies. Within this subgroup, 260 colonoscopies were needed to detect one case of CRC among patients with alarm symptoms, compared with 415 colonoscopies among those without alarm symptoms. In contrast, in patients with concordant positive FIT results, only five colonoscopies were needed to find one CRC.

When looking at the number needed to scope to find one advanced neoplasia for the whole study population, nine colonoscopies were needed. This number was higher among patients without alarm symptoms, where 13 colonoscopies were needed to detect one case of advanced neoplasia. The number needed to scope to find one advanced neoplasia was higher in the subgroup with concordant negative FIT results, at 23 colonoscopies. Within this subgroup, 26 colonoscopies were needed to detect one case of CRC among patients with alarm symptoms, compared with 21 colonoscopies among those without alarm symptoms. In patients with concordant positive FIT results, only two colonoscopies were needed to find one case of advanced neoplasia.

## Discussion

This prospective multicenter cohort study investigated the performance of two-sample FIT in ruling out the presence of relevant lesions in symptomatic patients. The highest NPV and sensitivities were reached with two-sample FIT using any detectable hemoglobin as the cutoff value. For advanced neoplasia, this resulted in an NPV of 95.6% with a sensitivity of 71.7%. For CRC alone, it even resulted in an NPV of 99.7% with a sensitivity of 93.9%. Both the use of one-sample FIT and increasing the cutoff level in two-sample FIT led to a decrease in NPV and sensitivity for all relevant lesions, and thus an increase in missed lesions. It did lead to an increase in PPV and specificity, but this is less important in a symptomatic population who currently all undergo diagnostic colonoscopy. Using two-sample FIT holds significant potential for guiding the need for colonoscopy in symptomatic patients, particularly given its minimal burden for patients and the health care system. Concordant negative results from two-sample FIT were found in up to 71% of patients. However, not offering colonoscopy to all these patients seems too rigorous as a substantial proportion of relevant lesions was still missed by two-sample FIT, including 28.3% of all advanced neoplasia. Critical appraisal of each patient is warranted to assess the applicability of two-sample FIT in averting colonoscopy.

A prior study comprising 2637 symptomatic patients who completed two-sample FIT showed an NPV of 98.1% and a sensitivity of 81.6% for advanced neoplasia 8 . This study used a cutoff level of 10 µg Hb/g feces with the use of the HM-JACK method (Hitachi Chemical Diagnostics, Mountain View, California, USA) 8 . We hypothesized that lowering the cutoff level to the lowest limit of detection would further increase sensitivity, and our study did indeed show an increase in sensitivity and NPV when lowering the cutoff value. However, even at any detectable hemoglobin cutoff value, sensitivity of two-sample FIT for advanced neoplasia was only 71.7%. This could be due to the lack of standardization of FIT tests, meaning that equal quantities of hemoglobin per gram feces will not result in equal results in different FIT tests. Therefore, it is important to keep in mind that thresholds cannot be directly transferred between studies without regard to the specific method used. A study performed by the International Federation of Clinical Chemistry and Laboratory Medicine working group on FIT showed that the Sentinel method used in our study had lower performance results when compared with the HM-JACK method 11 . It is also important to investigate which other factors can cause differences in two-sample FIT performance between different study populations. The population investigated in the Deprez et al. study 11 comprised patients with suspected CRC or urgent priority referrals, whereas our study population comprised all patients referred for colonoscopy because of symptoms or anemia. It is possible that the performance of two-sample FIT in ruling out advanced neoplasia is more reliable in patients with high-risk symptoms, as these lesions tend to be more advanced and therefore have a higher probability of shedding blood into the stool. This assumption corresponds to our analyses, where colonoscopy referral for the alarm symptoms of rectal blood loss and/or anemia resulted in a higher sensitivity for advanced neoplasia using two-sample FIT compared with the sensitivity found for the whole study population. Sensitivity for advanced neoplasia was 84.6% in the subgroup with alarm symptoms, which is higher than the sensitivity of 81.6% for advanced neoplasia found in the previously mentioned study 8 , confirming the benefit of lowering the cutoff value in our study results.

Applying two-sample FIT to rule out advanced neoplasia seems most reliable in patients referred for colonoscopy because of the alarm symptoms of rectal blood loss and/or anemia. However, this is also the subgroup harboring the highest proportion of relevant lesions in our study. In addition, a previous meta-analysis investigating the diagnostic accuracy of alarm features identified rectal blood loss and anemia as symptoms with the highest risk and therefore mandating colonoscopy 10 . It can be debated whether two-sample FIT should be applied to this subgroup to evaluate whether colonoscopy can be averted, or whether this subgroup should be referred for colonoscopy regardless of other tests. In that case, two-sample FIT could still be applied to patients without alarm symptoms. However, in patients without alarm symptoms, sensitivity for advanced neoplasia using two-sample FIT was low, and this subgroup accounted for the majority of advanced neoplasia cases missed following two negative FIT results.

Applying two-sample FIT to rule out other relevant lesions besides advanced neoplasia, such as advanced serrated polyps and colitis, also resulted in low sensitivities. NPV was still above 96% as the prevalence of these lesions was low. However, corresponding sensitivities were lower (33.3% for advanced serrated polyps and 61.4% for colitis) with broad confidence intervals, showing that the small proportion of false negatives represented a relatively large proportion of the patients with these relevant lesions. Two negative FITs missed a substantial proportion of advanced serrated polyps (66.7%) and colitis (38.6%). However, two-sample FIT did show potential for detecting colitis suspicious for IBD, with a sensitivity of 83.3%.

Concordant negative FIT results increased the number needed to scope to find one CRC from 29 colonoscopies to 338 colonoscopies. In addition, the negative likelihood ratio for two-sample FIT in ruling out relevant lesions was below 0.1 for CRC. The negative likelihood ratio is a value considered to provide strong evidence for ruling out a diagnosis. Therefore, the use of two-sample FIT may be considered in symptomatic patients when the primary goal is to rule out the presence of CRC. It should be noted that for patients without alarm symptoms, one-sample FIT could also be used for this purpose, as two-sample FIT did not increase the sensitivity and NPV for CRC within this subgroup.

For all other relevant lesions, two-sample FIT was better than one-sample FIT in ruling out relevant lesions, but still missed too many lesions for it to be used as a single determinant for the decision to avert colonoscopy. However, it could play a role in determining the need for colonoscopy in individual patients when applying it together with other factors, such as age, presence of alarm symptoms, medical history, eligibility to participate in a CRC screening program, and prior colonoscopies.

In this prospective multicenter cohort study, 949 symptomatic patients referred for colonoscopy completed two-sample FIT beforehand. Patients with all possible symptoms or anemia were eligible for inclusion, making it possible to assess differences in two-sample FIT performance in different subgroups of symptomatic patients. However, our study does have some limitations. First, in the main analyses on the performance of two-sample FIT versus one-sample FIT in ruling out relevant lesions, the 95%CIs were narrow for NPVs but wider for sensitivity; therefore, the sensitivities for different lesions should be interpreted with more caution. Second, subsequent pathology was not applied to all lesions found at colonoscopy, for example due to polyps lost during colonoscopy or referral to a nonparticipating endoscopy center for removal of large polyps. This could have led to an underestimation of relevant lesions in our study population. Third, it was unknown whether patients underwent prior colonoscopy. Prior colonoscopy can lower the risk of relevant lesions, which could have potentially biased our results. Fourth, all patients performed two-sample FIT making it difficult to directly compare these results with one-sample FIT; data for one-sample FIT were taken from the first FIT in the current study. Fifth, it could be debated whether FIT provides added value for patients with rectal blood loss, assuming that the patients reliably noticed blood in their stool. However, in our study population, 220 out of 358 patients with rectal blood loss had concordant negative results from two-sample FIT. Sixth, it is important to note that due to the lack of standardization of FIT tests, equal quantities of hemoglobin per gram feces will not result in equal results in different FIT tests 11 . Therefore, results from our study cannot be transferred directly to other patient populations without regard to the specific method used. Absolute FIT values as well as the lowest limit of detection could be different when using other methods for FIT testing. FIT method W results are provided in the Supplementary material. When applying this method in clinical practice it is important to note that FIT method W has a different limit of detection and uses different reference standards. Nevertheless, it is safe to speculate that, regardless of the FIT testing method used, the most sensitive cutoff will still be the lowest limit of detection and two-sample FIT will still have a higher sensitivity and NPV than one-sample FIT.

Prior guidelines have mostly focused on the use of FIT in identifying patients with symptoms suspicious for CRC that require quick referral for colonoscopy 3 4 . Our study investigated the potential of two-sample FIT in taking this a step further by possibly ruling out relevant lesions and thus averting colonoscopy. Using the lowest limit of fecal hemoglobin detection resulted in the highest NPV and sensitivity and thus the lowest number of missed lesions, which is desirable in symptomatic patients who currently all undergo colonoscopy. Up to 71% of symptomatic patients had concordant negative FIT results. However, not offering colonoscopy to all these patients seems too rigorous as a substantial proportion of relevant lesions was still missed by two-sample FIT, even in patients without alarm symptoms. Two-sample FIT seems reliable for the purpose of ruling out CRC. However, for all other lesions, two-sample FIT cannot be used as a single determinant for the decision to avert colonoscopy. Future research should focus on developing a clinical decision tool to guide clinicians in determining the need for colonoscopy in symptomatic patients, incorporating two-sample FIT alongside other determinants such as age, presence of alarm symptoms, medical history, and prior colonoscopies.

## Supplementary Material

Supplementary Material Zusatzmaterial
