# Supplementary material for: Two-sample fecal immunochemical testing as a tool to avert colonoscopy in symptomatic patients: a prospective multicenter cohort study
Source: Endoscopy. 2025 Aug 25;58(2):174–83. doi: 10.1055/a-2650-0664 (PMC12829320; doi:10.1055/a-2650-0664)
Supplement: Supplementary file 1 — Supplementary Material [file 10-1055-a-2650-0664_26659794.pdf]

Supplementary material

Two-sample fecal immunochemical testing as a tool to avert colonoscopy in symptomatic patients: a prospective multicenter cohort study

Sarah Moen*[1], Pieter H.A. Wisse*[1], Fleur E. Marijnissen[1], Hannah A.C. Raab[1], Iris Lansdorp-Vogelaar[2], Jeroen M. Jansen[3], Merel M. Tielemans[4], Ivonne Leeuwenburgh[5], Leonieke M.M. Wolters[6], Lieke Hol[7], Pieter ter Borg[8], Frank C. Bekkering[9], Sanna A. Mulder[10], Ingrid Schot[11], Marieke Frasa[12], Marc Thelen[13,14], Anneke J. van Vuuren[1], Manon C.W. Spaander[1]

 © Thieme. All rights reserved.

Thieme

Table 1s Performance of two-sample FIT and one-sample FIT in detecting relevant lesions at different cutoff values (FOB Gold set H method).

	**Two-sample FIT**				**One-sample FIT**			
	Sensitivity	***Specificity***	***PPV***	***NPV***	***Sensitivity***	***Specificity***	***PPV***	***NPV***
**Any detectable Hb**								
Advanced neoplasia	71.7%	76.5%	27.7%	95.6%	63.2%	82.6%	31.3%	94.7%
CRC	93.9%	73.5%	11.3%	99.7%	87.9%	79.8%	13.6%	99.5%
Advanced adenoma	62.7%	74%	17.2%	95.9%	53.3%	80.1%	18.7%	95.2%
Advanced serrated polyps	33.3%	71.3%	4%	96.7%	24.2%	77.5%	3.7%	96.6%
Colitis	61.4%	73.2%	12.8%	96.7%	52.6%	79.4%	14%	96.3%
Colitis suspicious for IBD	83.3%	72.9%	9.1%	99.3%	76.7%	79.2%	10.7%	99%
Colitis not IBD related	37%	71.4%	3.6%	97.5%	25.9%	77.5%	3.3%	97.3%
**Cutoff value: 10 µg/g**								
Advanced neoplasia	69.8%	81.1%	31.8%	95.5%	59.4%	86.1%	35%	94.4%
CRC	93.9%	77.9%	13.3%	99.7%	87.9%	83.5%	16.1%	99.5%
Advanced adenoma	60%	78.5%	19.3%	95.8%	48%	83.5%	20%	94.9%
Advanced serrated polyps	27.3%	75.5%	3.9%	96.6%	21.2%	81.1%	3.9%	96.6%
Colitis	57.9%	77.6%	14.2%	96.6%	45.6%	82.7%	14.4%	96%
Colitis suspicious for IBD	80%	77.3%	10.3%	99.2%	70%	82.7%	11.7%	98.8%
Colitis not IBD related	33.3%	75.7%	3.9%	97.5%	18.5%	81%	2.8%	97.1%
**Cutoff value: 45 µg/g**								
Advanced neoplasia	63.2%	86.5%	37%	94.9%	55.7%	89.8%	40.7%	94.2%
CRC	87.9%	83.4%	16%	99.5%	87.9%	87.3%	20%	99.5%
Advanced adenoma	53.3%	83.9%	22.1%	95.4%	42.7%	87.1%	22.1%	94.7%
Advanced serrated polyps	21.2%	81%	3.9%	96.6%	18.2%	84.8%	4.1%	96.6%
Colitis	47.4%	82.7%	14.9%	96.1%	43.9%	86.5%	17.2%	96%
Colitis suspicious for IBD	73.3%	82.7%	12.2%	99%	70%	86.5%	14.5%	98.9%
Colitis not IBD related	18.5%	80.9%	2.8%	97.1%	14.8%	84.7%	2.8%	97.1%

CRC = colorectal cancer; FIT = fecal immunochemical Test; PPV = positive predictive value; NPV = negative predictive value; IBD = inflammatory bowel disease.

** Any detectable Hb corresponds to the lower limit of detection of 1,7 µg/g.*

** Two-sample FIT was classified as positive when FIT1 and/or FIT2 was positive.*

Table 2s Performance of two-sample FIT and one-sample FIT in detecting relevant lesions at different cutoff values (FOB Gold set W method).

	**Two-sample FIT**				**One-sample FIT**			
**Any detectable Hb**	***Sensitivity***	***Specificity***	***PPV***	***NPV***	***Sensitivity***	***Specificity***	***PPV***	***NPV***
Advanced neoplasia	68.9%	78.9%	29.1%	95.3%	61.3%	83.6%	32%	94.5%
CRC	90.9%	75.9%	12%	99.6%	84.8%	80.9%	13.8%	99.3%
Advanced adenoma	60%	76.4%	17.9%	95.7%	52%	81.2%	19.2%	95.2%
Advanced serrated polyps	27.3%	73.6%	3.6%	96.6%	18.2%	78.5%	3%	96.4%
Colitis	56.1%	75.4%	12.7%	96.4%	49.1%	80.4%	13.8%	96.1%
Colitis suspicious for IBD	80%	75.3%	9.6%	99.1%	73.3%	80.3%	10.8%	98.9%
Colitis not IBD related	29.6%	73.6%	3.2%	97.3%	22.2%	78.6%	3%	97.2%
**Cut-off value: 10 µg/g**	***Sensitivity***	***Specificity***	***PPV***	***NPV***	***Sensitivity***	***Specificity***	***PPV***	***NPV***
Advanced neoplasia	58.5%	89.3%	40.8%	94.5%	49.1%	91.8%	43%	93.5%
CRC	84.8%	86.5%	18.4%	99.4%	81.8%	89.7%	22.3%	99.3%
Advanced adenoma	48%	86.7%	23.7%	95.1%	36%	89.2%	22.3%	94.2%
Advanced serrated polyps	21.2%	84.2%	4.6%	96.7%	15.2%	87.3%	4.1%	96.6%
Colitis	45.6%	85.9%	17.1%	96.1%	38.6%	88.9%	18.2%	95.8%
Colitis suspicious for IBD	70%	85.7%	13.8%	98.9%	60%	88.8%	14.9%	98.6%
Colitis not IBD related	18.5%	84.1%	3.3%	97.2%	14.8%	87.3%	3.3%	97.2%
**Cut-off value: 45 µg/g**	***Sensitivity***	***Specificity***	***PPV***	***NPV***	***Sensitivity***	***Specificity***	***PPV***	***NPV***
Advanced neoplasia	44.3%	92.9%	43.9%	93%	37.7%	95.1%	49.4%	92.4%
CRC	75.8%	91%	23.4%	99%	75.8%	93.9%	30.9%	99.1%
Advanced adenoma	32%	90.5%	22.4%	93.9%	22.7%	92.7%	21%	93.3%
Advanced serrated polyps	15.2%	88.9%	4.7%	96.7%	15.2%	91.7%	6.2%	96.8%
Colitis	35.1%	90.2%	18.7%	95.6%	33.3%	93%	23.5%	95.6%
Colitis suspicious for IBD	56.7%	90.2%	15.9%	98.5%	53.3%	92.9%	19.8%	98.4%
Colitis not IBD related	11.1%	88.7%	2.8%	97.1%	11.1%	91.5%	3.7%	97.2%

CRC = colorectal cancer; FIT = fecal immunochemical Test; PPV = positive predictive value; NPV = negative predictive value; IBD = inflammatory bowel disease.

** Any detectable Hb corresponds to the lower limit of detection which for the FIT method W was 2.5 µg/g.*

** Two-sample FIT was classified as positive when FIT1 and/or FIT2 was positive.*

Thieme

Table 3s Performance of two-sample FIT versus one-sample FIT in detecting relevant lesions for subgroups with and without alarm symptoms.

	**N**	**Two-sample FIT**				**One-sample FIT**			
**Indication alarm symptoms**	423	***Sensitivity***	***Specificity***	***PPV***	***NPV***	***Sensitivity***	***Specificity***	***PPV***	***NPV***
Advanced neoplasia	65	84.6%	69.8%	33.7%	96.2%	75.4%	77.4%	37.7%	94.5%
CRC	26	96.2%	65.2%	15.3%	99.6%	88.5%	73%	17.7%	99%
Advanced adenoma	41	78.0%	65.7%	19.6%	96.5%	68.3%	73.3%	21.5%	95.6%
Advanced serrated polyps	17	35.3%	61.3%	3.7%	95.8%	35.3%	69.5%	4.6%	96.2%
Colitis	31	67.7%	63.8%	12.9%	96.2%	64.5%	71.9%	15.4%	96.2%
Colitis suspicious for IBD	22	86.4%	64.1%	11.7%	98.8%	81.8%	72.1%	13.8%	98.6%
Colitis not IBD related	9	22.2%	61.1%	1.2%	97.3%	22.2%	69.1%	1.5%	97.6%
**Indication other symptoms**	526	***Sensitivity***	***Specificity***	***PPV***	***NPV***	***Sensitivity***	***Specificity***	***PPV***	***NPV***
Advanced neoplasia	41	51.2%	81.4%	18.9%	95.2%	43.9%	86.4%	21.4%	94.8%
CRC	7	85.7%	79.8%	5.4%	99.8%	85.7%	85%	7.1%	99.8%
Advanced adenoma	34	44.1%	80.5%	13.5%	95.4%	35.3%	85.4%	14.3%	95%
Advanced serrated polyps	16	31.3%	79.2%	4.5%	97.3%	12.5%	83.9%	2.4%	96.8%
Colitis	26	53.8%	80.6%	12.6%	97.1%	38.5%	85.2%	11.9%	96.4%
Colitis suspicious for IBD	8	75.0%	79.7%	5.4%	99.5%	62.5%	84.7%	6%	99.3%
Colitis not IBD related	18	44.4%	79.7%	7.2%	97.6%	27.8%	84.4%	6%	97.1%

CRC = colorectal cancer; FIT = fecal immunochemical test; PPV = positive predictive value; NPV = negative predictive value; IBD = inflammatory bowel disease.

** Any detectable Hb corresponds to the lower limit of detection of 1,7 µg/g.*

** Two-sample FIT was classified as positive when FIT1 and/or FIT2 was positive.*

 © Thieme. All rights reserved.

Thieme

Table 4s Numbers needed to scope to find one CRC or one advanced neoplasia depending on presence of alarm symptoms and results from two-sample FIT.

	Total N	N with CRC	NNTS to find one CRC	N with AN	NNTS to find one AN
All participants	949	33	28.8	106	9.0
Indication alarm symptoms	423	26	16.3	65	6.5
Indication other symptoms	526	7	75.1	41	12.8
All participants with two negative FITs	675	2	337.5	30	22.5
Indication alarm symptoms	260	1	260	10	26
Indication other symptoms	415	1	415	20	20.8
All participants with discordant FITs	124	2	62	14	8.9
Indication alarm symptoms	65	2	32.5	8	8.1
Indication other symptoms	59	0	NA	6	9.8
All participants with two positive FITs	150	29	5.2	62	2.4
Indication alarm symptoms	98	23	4.3	47	2.1
Indication other symptoms	52	6	8.7	15	3.5

FIT = fecal immunochemical test; N = number; CRC = colorectal cancer; AN = advanced neoplasia; NNTS = numbers needed to scope.

Endoscopy | DOI 10.1055/a-2650-0664 | © Thieme. All rights reserved.
